# Use of Lipid-Lowering Medications and the Likelihood of Achieving Optimal LDL-Cholesterol Goals in Coronary Artery Disease Patients

**DOI:** 10.1155/2012/861924

**Published:** 2012-07-25

**Authors:** Dean G. Karalis, Brett Victor, Lilian Ahedor, Longjian Liu

**Affiliations:** ^1^Cardiology Consultants of Philadelphia, Philadelphia, PA 19107, USA; ^2^Division of Cardiology, Drexel University College of Medicine, Philadelphia, PA 19107, USA; ^3^Department of Epidemiology and Biostatistics, Drexel University School of Public Health, Philadelphia, PA 19107, USA

## Abstract

*Background*. In clinical practice, most coronary artery disease patients are not achieving their recommend LDL-cholesterol goal of <70 mg/dL. Methods. We conducted a retrospective analysis of outpatient electronic health records and the most recent lipid profile, lipid-lowering medications and doses were collected. Results. We identified 9950 coronary artery disease patients. Only 37% on a statin alone achieved an LDL-cholesterol of <70 mg/dL, and most were on moderate-to-high-potency statins. The intensity of statin therapy did not improve LDL-cholesterol goal attainment. Among patients on combination therapy, 41% on statin plus ezetimibe and 46% on statin plus niacin achieved an LDL-cholesterol of <70 mg/dL (*P* = 0.01 and <0.0001 versus statin alone). If patients were switched to a high-potency statin LDL-cholesterol goal attainment of <70 mg/dL would increase to 46% and would increase up to 72% with combination therapy. Conclusions. Most coronary artery disease patients in clinical practice do not attain an LDL-cholesterol of <70 mg/dL, even among patients on high potency statins. The combination of statin plus either ezetimibe or niacin is the most effective regimen to achieve an LDL-cholesterol of <70 mg/dL, however, these drug combinations are used infrequently in clinical practice.

## 1. Introduction

Coronary artery disease (CAD) is the leading cause of death and disability in both men and women in the United States [1]. Patients with known CAD are considered at high or very high cardiovascular risk and require aggressive modification of all their risk factors [2]. Several major clinical trials with statin therapy in patients with CAD have shown that lowering low-density lipoprotein (LDL) cholesterol can reduce cardiovascular events and the lower the LDL-cholesterol achieved the lower the cardiovascular risk [3–7]. In the Cholesterol Treatment Trialists' (CTT) Collaboration meta-analysis of individual data from over 170,000 patients in 26 randomized trials of statins, coronary mortality was reduced by just over one-fifth per 1.0 mmol/L (or 39 mg/dL) reduction in LDL-cholesterol with no evidence of any threshold within the range of cholesterol studied [8]. In a recent analysis from the JUPITER (Justification for the Use of Statins in Prevention: an Intervention Trial of Evaluating Rosuvastatin) trial, individuals who achieved an LDL-cholesterol of <50 mg/dL had a lower cardiovascular risk than those with higher on-treatment levels of LDL-cholesterol [9].

Based on a wealth of clinical trial evidence, current guidelines now recommend more aggressive LDL-cholesterol targets. Recent guidelines from the United States recommend that an LDL-cholesterol goal of <70 mg/dL is reasonable in high and very high risk patients such as those with CAD [2, 10, 11]. The American Diabetes Association and the American College of Cardiology identify adults with clinical CAD as being at the highest risk and recommend an LDL-cholesterol treatment goal of <70 mg/dL for these patients [12]. The new ESC/EAS Guidelines for the management of dyslipidaemias also recommend an LDL-cholesterol goal of <70 mg/dL for individuals with known cardiovascular disease [13].

Despite current guidelines, several studies have shown that most CAD patients do not achieve their recommended LDL-cholesterol goal of <70 mg/dL. We have previously reported that in clinical practice only about one-third of CAD patients achieve an LDL-cholesterol goal of <70 mg/dL [14]. Other observational studies have shown a similar low rate of attaining an LDL-cholesterol goal of <70 mg/dL in CAD patients [15–17]. There have been a number of barriers identified as to why CAD patients fail to achieve their optimal LDL-cholesterol goals in clinical practice [18]. These include patient noncompliance, physician nonadherence to current guidelines, intolerance to lipid-lowering therapy, and the lack of affordable and effective lipid-lowering medications to allow patients to achieve their more aggressive LDL-cholesterol goals.

 The purpose of this study was to evaluate in a real-world setting what lipid-lowering medications are prescribed, the potency of statin therapy and the frequency of combination therapy being used in clinical practice to treat patients with CAD. It was our intention to determine if one barrier to achieving an LDL-cholesterol of <70 mg/dL was the underutilization of lipid-lowering therapy in CAD patients by physicians in clinical practice.

## 2. Methods

The study site was Cardiology Consultants of Philadelphia, a large cardiology subspecialty practice in the Philadelphia area. Using an electronic medical record, we identified 23,408 patients with a history of CAD who had been seen in one of our outpatient offices over a 12-month period from September 2008 to September 2009. Patients were excluded if they did not have a complete lipid panel within the electronic medical record flow sheet dated from within the study period or within 6 months of their last office visit. In patients with more than one complete lipid panel, the most recent lipid panel was used. The use of lipid-lowering medications and their doses was noted. We identified 9950 CAD patients who met the study criteria. The study was approved by the Institutional Review Board of Drexel University College of Medicine.

Statin potency was categorized as low, moderate, or high potency. High-potency statins included atorvastatin (40 mg or 80 mg), rosuvastatin (20 mg or 40 mg), or simvastatin 80 mg. Moderate-potency statins included atorvastatin (10 mg or 20 mg), rosuvastatin (10 mg), simvastatin 40 mg, lovastatin 80 mg, or pravastatin 80 mg. All other statin doses were considered as low-potency statins. We used the following assumptions to predict LDL-cholesterol goal attainment if patients were switched to a more potent statin or if another non-statin LDL-cholesterol lowering drug was added to their statin therapy. For each change from a low to moderate potency statin or from a moderate to high potency statin we assumed a 6% additional decrease in LDL-cholesterol. We assumed a 15% additional decrease in LDL-cholesterol when a non-statin LDL-lowering drug was added to a patient on statin therapy alone. Non-statin LDL-lowering drugs included ezetimibe, bile-acid sequestrants or niacin. We assumed a 10% intolerance rate for any change to a more potent statin or for all non-statin LDL-lowering drugs.

For statistical analysis we examined mean differences between two groups using the *t*-test, and among three groups using analysis of variance, and examined differences in the prevalence of goal attainment across different groups using the chi-square test. All data analyses were conducted using SAS software version 9.2 (SAS Institute Inc., Cary, NC 27513). The level of significance was set at a two-sided *P*-value of ≤0.05.

## 3. Results

Among the 9950 CAD patients, a total of 5885 patients (59%) were treated with statin therapy alone, 2730 patients (27%) were treated with a statin in combination with another lipid-lowering drug, 389 (4%) were not on a statin but taking another non-statin lipid-lowering drug, and 946 (10%) were on no lipid-lowering therapy (Table 1). Among patients treated with statin therapy alone, 2174 patients (37%) achieved an LDL-cholesterol goal of <70 mg/dL (Table 2). Most patients were on moderate-potency statin therapy (41%), while the number of patients on high- and low-potency statin therapy was similar; 29% and 30%, respectively.

**Figure 1:**
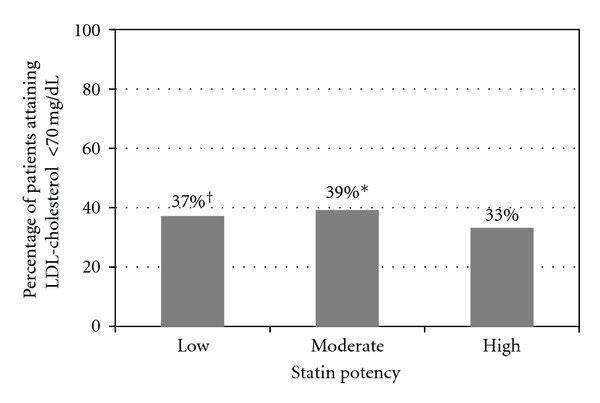
Percentage of patients achieving an LDL-cholesterol of <70 mg/dL based on the potency of their statin therapy. Fewer patients achieved an LDL-cholesterol goal of <70 mg/dL on high-potency statins compared to those on moderate or low-potency statins (*P < 0.0001 and ^†^ *P* < 0.01 compared to high potency statin therapy).

The mean achieved LDL-cholesterol level in the high potency statin group was significantly higher (81.4 mg/dL ± 27.0) when compared to the moderate-potency group (78.7 mg/dL ± 26.7); *P* < 0.01 and to the low-potency group (79.8 mg/dL ± 26.5); *P* = 0.02. Fewer patients on high-potency statins achieved their LDL-cholesterol goal of <70 mg/dL (33%) compared to patients on moderate potency statins (39%); *P* < 0.0001, and to patients on low potency statins (37%); *P* < 0.01. Only 37% of patients taking any dose of atorvastatin or rosuvastatin attained an LDL-cholesterol goal of <70 mg/dL. There was no relationship between increasing statin dose and improved LDL-cholesterol goal attainment (Figure 1).

**Figure 2:**
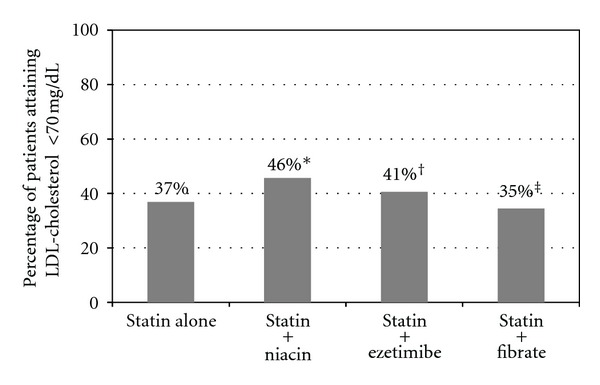
Percentage of patients achieving an LDL-cholesterol of <70 mg/dL on statin therapy alone compared to statin therapy in combination with another lipid-lowering drug. More patients achieved an LDL-cholesterol goal of <70 mg/dL with statin plus niacin (*P < 0.001) and with statin plus ezetimibe (^†^ *P* = 0.01) compared with statin therapy alone. The combination of a statin and fibrate did not improve LDL-cholesterol goal attainment compared to statin therapy alone (^‡^ *P* = 0.23).

Patients treated with a combination of statin and niacin were more likely to attain an LDL-cholesterol of <70 mg/dL (46% versus 37%; *P* < 0.0001) compared to patients treated with statin therapy alone as were patients treated with a combination of statin and ezetimibe (41% versus 37%; *P* = 0.01, Figure 2). However, only 9% of patients were treated with a combination of statin and niacin and only 14% of patients were on a combination of statin and ezetimibe. Patients treated with statin and a fibrate did not improve their LDL-cholesterol goal attainment when compared to patients treated with a statin alone (35% versus 37%; *P* = 0.23). The number of patients treated with a bile acid sequestrant or high-dose fish oil in combination with a statin was small; both <1%. Patients on no statin but treated with other lipid lowering drugs were less likely to achieve their LDL-cholesterol goals compared to patients on statin therapy alone (18% versus 37%; *P* < 0.0001) as were patients on no lipid-lowering therapy (20% versus 37%; *P* < 0.0001).

Among patients taking a statin in combination with another lipid-lowering drug, most were taking only one additional drug (90%), while 10% were taking 2 or more lipid lowering drugs plus statin therapy. Among patients taking other lipid-lowering therapy, but not on a statin, 69% were on only one non-statin drug while 31% were on 2 or more non-statin drugs.

 It was estimated that if statin alone patients who had not attained their LDL-cholesterol goal of <70 mg/dL on a low or moderate potency statin were switched to a high-potency statin, assuming 90% tolerance of high-dose statin therapy, LDL-cholesterol goal attainment of <70 mg/dL would have increased from 37% to 46%; *P* < 0.01. If these patients were started on a non-statin LDL-cholesterol lowering drug instead of switching to a high-potency statin, and again assuming 90% tolerance to their non-statin lipid-lowering therapy, LDL-cholesterol goal attainment of <70 mg/dL would have increased from 37% to 60%; *P* < 0.01. More CAD patients would have achieved an LDL-cholesterol of <70 mg/dL by adding another non-statin LDL-lowering drug as compared to switching to a high-potency statin (60% versus 46%; *P* < 0.01). If all statin-alone patients were on a high-potency statin in combination with another non-statin LDL-cholesterol-lowering drug, LDL-cholesterol goal attainment of <70 mg/dL would have increased to 63%. If all patients were taking a high-potency statin and 2 additional non-statin LDL-cholesterol-lowering drugs, LDL-cholesterol goal attainment of <70 mg/dL would increase further to 72%.

## 4. Discussion

 Our study demonstrates that, among CAD patients treated with statin therapy alone, only about one in three are achieving an LDL-cholesterol goal of <70 mg/dL, despite the fact that most are on moderate-to-high-potency statins. Although previous studies have also shown that few high-risk CAD patients attain an LDL-cholesterol of <70 mg/dL, few studies such as ours have evaluated the attainability of this more aggressive LDL-cholesterol goal as it relates to the choice of lipid lowering therapy. Kauffman and colleagues [19] found that, among 7427 CAD patients, 43% achieved an LDL-cholesterol goal of <70 mg/dL and similar to our findings, the majority of patients were receiving moderate to high-potency statin monotherapy. In addition, patients taking high-potency statin monotherapy were less likely to attain an LDL-cholesterol goal of <70 mg/dL compared to patients taking less potent statins. We observed the same finding in our study. This paradoxical finding may be explained by the fact that, in clinical practice, managed care plans often mandate the use of a generic statin first and only when patients fail to achieve their desired LDL-cholesterol goal are the use of higher potency nongeneric statins allowed. It is likely that patients with the most difficult to treat lipids are the ones that are treated with the highest doses of the most potent statins, such as atorvastatin or rosuvastatin. However, even if all CAD patients could be switched to a high-potency statin, less than one-half of these high-risk patients would achieve an LDL-cholesterol goal of <70 mg/dL.

Statins remain the cornerstone of LDL-cholesterol lowering, however, in clinical practice, most CAD patients will not be able to achieve an LDL-cholesterol goal of <70 mg/dL without adding another LDL-cholesterol-lowering drug to statin therapy. In our study, as in previously published reports, the number of patients on a statin in combination with another LDL-cholesterol-lowering drug was low despite the evidence for the safety and efficacy of combination lipid-lowering therapy [20–24]. Numerous studies have shown that the combination of a statin plus ezetimibe is superior to statin monotherapy with regards to LDL-cholesterol-lowering and attaining an LDL-cholesterol goal of <70 mg/dL [25–27]. Ezetimibe will lower LDL-cholesterol by an additional 15 to 20% when added to statin therapy. However, few patients in our study were on ezetimibe in combination with a statin. This may be related to the lack of cardiovascular outcome data with ezetimibe in combination with a statin compared to statin therapy alone, as well as to some safety concerns regarding ezetimibe that have been raised from other clinical trials. However, in the recent Study of Heart and Renal Protection (SHARP), cholesterol lowering with a combination of simvastatin and ezetimibe reduced major atherosclerotic events by 17% compared to placebo in a population of chronic kidney disease patients [28]. In addition, there was a significant 25% reduction in nonhemorrhagic stroke and a significant 27% reduction in coronary revascularization in this population without any evidence for excess risk of hepatitis, gallstones, or cancer in ezetimbe-treated patients. This should reassure physicians about the safety and efficacy of ezetimibe and its role in clinical practice in attaining optimal LDL-cholesterol levels in patients who have not achieved their LDL-cholesterol goal despite statin monotherapy.

Although niacin is thought mostly as a high-density lipoprotein (HDL) raising drug, in higher doses niacin will lower LDL-cholesterol by approximately 15 to 20% when added on to statin therapy [29, 30]. As with ezetimibe, niacin was used infrequently in our study in combination with a statin, yet both of these combinations led to significantly greater attainment of an LDL-cholesterol goal of <70 mg/dL than did statin monotherapy. While the negative findings from the AIM-HIGH (Atherothrombosis Intervention in Metabolic Syndrome with Low HDL/High Triglycerides: Impact on Global Health Outcomes) trial [31] may dissuade physicians from using niacin more often in clinical practice, niacin may play a role to lower LDL-cholesterol levels in statin intolerant patients. Niacin may also be beneficial in patients on statin monotherapy who have not achieved their LDL-cholesterol goal especially in the setting of a low HDL-cholesterol or high levels of triglycerides. Bile acid sequestrants also provide additional LDL-cholesterol lowering, however, in our study these drugs were used in less than one percent of our CAD patients.

It must be emphasized that statins are the drug of choice to lower LDL cholesterol in patients with CAD and the addition of another LDL-lowering drug to a statin should only be considered in patients not at their LDL-cholesterol goal on a maximally tolerated dose of statin or for those patients who are statin intolerant. Despite the abundant outcome data for statins in CAD patients, there is no large prospective outcome study that had demonstrated the benefits of combination lipid-lowering therapy over statin therapy alone. However, many CAD patients are unable to achieve their LDL-cholesterol goal even with a high-potency statin and statin intolerance remains a significant problem in real world clinical practice. In large randomized clinical trials with statin therapy, the incidence of myalgias is generally low and approximately 2 to 4% [3–6]. However, data from observational studies have reported a much higher incidence of myalgias from statin therapy. In one study from a large managed care database, 8 to 9% of patents reported myalgias that led to discontinuing their statin therapy [32]. In our study, the number of patients who were not on a statin was high at 13%. Other studies have shown an even higher number of high-risk CAD patients who are not taking a statin. In a recent analysis from a large managed-care claims database in the United States, almost 30% of patients with coronary heart disease or atherosclerotic vascular disease were on no lipid-lowering therapy [33]. Despite the clear benefit in reducing cardiovascular events afforded by statin therapy, many high-risk patients are not treated with statin therapy. This reality plus the finding that many patients on high potency statins still do not achieve an LDL-cholesterol of <70 mg/dL makes achieving optimal LDL-cholesterol goals difficult with statin therapy alone.

In our study, we made assumptions to estimate how many patients already tolerating statin therapy could achieve an LDL-cholesterol goal of <70 mg/dL if they were switched to a high-potency statin in combination with one or two additional non-statin LDL-lowering drugs. We used a liberal estimate of 10% for intolerance to a high-potency statin or to a non-statin LDL-lowering drug. In such a scenario attainment of an LDL-cholesterol goal of <70 mg/dL increased from 46% to 63% when patients on a high-potency statin had one additional non-statin LDL-lowering drug added and to 72% if 2 additional non-statin LDL-lowering drugs were added. In real-world clinical practice physicians need to use combination therapy more often if an LDL-cholesterol goal of <70 mg/dL is to be achieved.

The dilemma that physicians face in clinical practice is that no large cardiovascular outcome study has shown a benefit of combination lipid lowering therapy over statin therapy alone, yet most high-risk patients cannot achieve their recommended LDL-cholesterol goal without combination lipid-lowering therapy. This poses a significant challenge to the practicing clinician since more than one-half of CAD patients will not achieve their LDL-cholesterol goal of <70 mg/dL with statin therapy alone. The AIM-HIGH (Atherothrombosis Intervention in Metabolic Syndrome with Low HDL/High Triglycerides: Impact on Global Health Outcomes) trial [31] randomized 3414 with atherosclerotic cardiovascular disease and low levels of HDL-cholesterol to extended-release niacin, 1500 to 2000 mg a day, or placebo. All patients received simvastatin, 40 to 80 mg a day plus ezetimibe if needed to maintain an LDL-cholesterol of <80 mg/dL. The primary end point was a composite of coronary heart disease death and major cardiovascular events. No clinical benefit was seen with the addition of niacin during a 36-month follow-up period, however, the placebo group was well treated with a mean LDL-cholesterol level of 68 mg/dL by the end of the trial. The ACCORD (Action to Control Cardiovascular Risk in Diabetes) trial [34] randomized 5518 patients with type 2 diabetes to fenofibrate or placebo. All patients were also treated with open label simvastatin. The primary outcome was nonfatal myocardial infarction, stroke, or death from cardiovascular causes and patients were followed for a mean of 4.7 years. By the end of the study, there was no difference in LDL-cholesterol levels between groups and although triglyceride levels were reduced further in the fenofibrate group, the placebo group had normal triglyceride levels by the end of the study. The simvastatin-alone group was well treated with a mean LDL-cholesterol of <80 mg/dL. The combination of fenofibrate and simvastatin did not improve outcomes compared to simvastatin alone. In both the ACCORD and AIM-HIGH trials, the LDL-cholesterol levels in the placebo group were low and these studies do not adequately address the problem of how to treat the patient who is statin intolerant or whose LDL-cholesterol is not a goal despite statin therapy. Ongoing large outcome studies such as the HPS2-THRIVE comparing simvastatin plus niacin and laropiprant to simvastatin alone in CAD patients and IMPROVE-IT comparing the combination of ezitimibe and simvastatin to simvastatin alone in acute coronary syndrome patients will provide important information on the role of combination lipid-lowering therapy in the treatment of CAD patients.

As we await the results of these trials the practicing clinician should use statin therapy as the first choice for LDL-cholesterol-lowering in CAD patients. Other non-statin lipid lowering drugs should be used when the LDL-cholesterol and non-HDL cholesterol are not at goal despite statin therapy or in the statin-intolerant patient. Clinical studies and current guidelines support this recommendation. The SANDS (Stop Atherosclerosis in Native Diabetics Study) trial [35] randomized 499 American Indian men and women over the age of 40 with type 2 diabetes and no prior cardiovascular events to aggressive versus standard treatment for their lipids. The aggressive treatment group had an LDL-cholesterol goal of <70 mg/dL and a non-HDL cholesterol goal of <100 mg/dL while the standard treatment group had an LDL-cholesterol goal of <100 mg/dL and a non-HDL cholesterol goal of <130 mg/dL. Patients were treated with statin therapy and then as needed additional non-statin lipid lowering drugs were added to achieve the specified LDL and non-HDL cholesterol goals. The primary end point was progression of atherosclerosis as measured by common carotid artery intimal medial thickness (IMT). Patients were followed for 3 years. Compared with baseline, IMT regressed in the aggressive group and progressed in the standard group.

The strength of our study is that it represents real clinical practice. However, there are several limitations to our study. Our study was from a single site and only involved patients who were managed by a cardiologist. Our findings may not be generalizable to high-risk patients managed by primary care physicians or to CAD patients in other parts of the country. In addition, we only included patients who had a recent lipid profile in their electronic medical record and since patients without a recent lipid profile are more likely to be undertreated, we may have overestimated the actual number of patients who achieved their LDL-cholesterol goal. However, our findings are similar to other surveys that have reported that few CAD patients achieve optimal LDL-cholesterol levels with statin therapy alone. Furthermore, baseline lipid levels before treatment, specific information as to intolerance to lipid lowering therapy, and why specific patients were not on statin therapy, were not available from the electronic medical record.

## 5. Conclusions

 In conclusion, most CAD patients in real-world clinical practice do not attain an LDL-cholesterol of <70 mg/dL, even among patients on high-potency statins. The number of CAD patients on no statin is high. The combination of statin plus either ezetimibe or niacin is the most effective regimen to achieve an LDL-cholesterol of <70 mg/dL, however, these drug combinations are used infrequently in clinical practice. Statins remain the cornerstone of LDL-cholesterol-lowering in CAD patients, however, physicians need to consider using combination lipid-lowering therapy more often if an LDL-cholesterol goal of <70 mg/dL is to be realized in clinical practice. Finally, there is a need for novel lipid-lowering therapies, especially given the large number of high-risk patients not treated with statin therapy.

## Figures and Tables

**Table 1:** Lipid-lowering medications in the 9950 coronary artery disease patients.

Lipid-lowering medications	Patients
Total number of patients	9950
*Statin monotherapy*	5885 (59%)
High potency	1692 (17%)
Moderate potency	2398 (24%)
Low potency	1795 (18%)
*Combination t* *he* *ra* py*	2730 (27%)
Statin plus ezetimibe	1378 (14%)
Statin plus niacin	866 (9%)
Statin plus fibrate	650 (7%)
Statin plus fish oil	69 (<1%)
Statin plus bile acid sequestrant	43 (<1%)
*No statin*	1335 (13%)
*No statin but other lipid lowering t* *he* *ra* *py* ^†^	389 (4%)
Ezetimibe	179 (2%)
Niacin	113 (1%)
Fibrate	170 (2%)
Fish oil	11 (<1%)
Bile acid sequestrant	37 (<1%)
*No lipid-lowering therapy*	946 (10%)

^∗^Among patients on a statin plus another lipid-lowering medication, 276 patients were on more than one non-statin lipid-lowering drug.

^†^Among patients on no statin but another lipid-lowering medication, 121 patients were on more than one non-statin lipid-lowering drug.

**Table 2:** Patients attaining an LDL-cholesterol of <70 mg/dL according to their lipid lowering medications.

Lipid-lowering medications	Patients	LDL (mg/dL)	Patients attaining LDL <70
All Patients	9950	82.0 ± 29.2	3555 (36%)
*Statin monotherapy*	5885	79.8 ± 26.5	2174 (37%)
High potency	1692	81.4 ± 27.0	559 (33%)
Moderate potency	2398	78.7 ± 26.7	946 (39%)
Low potency	1795	79.8 ± 25.7	669 (37%)
Combination therapy*	2730		1119 (41%)
Any statin plus ezetimibe	1378	79.0 ± 28.6	559 (41%)
Any statin plus niacin	866	74.1 ± 24.7	396 (46%)
Any statin plus fibrate	650	81.3 ± 27.6	224 (35%)
*No statin*	1335	99.9 ± 35.7	262 (20%)
*No statin but other lipid-lowering therapy^†^*	389		70 (18%)
Ezetimibe	179	107.1 ± 33.1	20 (11%)
Niacin	113	96.3 ± 36.0	23 (20%)
Fibrate	170	94.3 ± 35.1	39 (23%)
*No lipid-lowering therapy*	946	99.5 ± 35.5	192 (20%)

^∗^Among patients on a statin plus another lipid-lowering drug 276 patients were on more than one non-statin lipid-lowering drug.

^†^Among patients on no statin but another lipid-lowering drug, 121 patients were on more than one non-statin lipid-lowering drug.

LDL-cholesterol values are expressed as mean ± SD.
